# Supplementary material for: Prediction of Protein–Protein Interaction Sites Using Convolutional Neural Network and Improved Data Sets
Source: Int J Mol Sci. 2020 Jan 11;21(2):467. doi: 10.3390/ijms21020467 (PMC7013409; doi:10.3390/ijms21020467)
Supplement: Supplementary file 1 [file ijms-21-00467-s001.zip › ijms-663638supplementary/Figure S1 Residue pairs contact propensities in protein-protein interface.docx]

**Figure S1.** Residue pairs contact propensities in protein-protein interface.
