# Supplementary material for: Prediction of Protein–Protein Interaction Sites Using Convolutional Neural Network and Improved Data Sets
Source: Int J Mol Sci. 2020 Jan 11;21(2):467. doi: 10.3390/ijms21020467 (PMC7013409; doi:10.3390/ijms21020467)
Supplement: Supplementary file 1 [file ijms-21-00467-s001.zip › ijms-663638supplementary/Table S3.docx]

**Table S3.** Accuracy and recall of our model in different threshold.

| **Threshold** | **Accuracy** | **Recall** |
| --- | --- | --- |
| 0.5 | 0.742 | 0.841 |
| 0.55 | 0.765 | 0.844 |
| 0.6 | 0.789 | 0.844 |
| 0.65 | 0.815 | 0.8378 |
| 0.7 | 0.847 | 0.824 |
| 0.75 | 0.887 | 0.797 |
| 0.8 | 0.936 | 0.730 |
| 0.85 | 0.974 | 0.632 |
| 0.9 | 0.990 | 0.567 |
| 0.95 | 0.997 | 0.523 |

For imbalanced samples that the number of positive samples is far less than that of negative samples, accuracy and recall will no longer be reliable, we have gave the values under different thresholds for reference.
