# Supplementary material for: Prediction of Protein–Protein Interaction Sites Using Convolutional Neural Network and Improved Data Sets
Source: Int J Mol Sci. 2020 Jan 11;21(2):467. doi: 10.3390/ijms21020467 (PMC7013409; doi:10.3390/ijms21020467)
Supplement: Supplementary file 1 [file ijms-21-00467-s001.zip › ijms-663638supplementary/Table S4.docx]

**Table S3.** Amino acid encoding.

| **No.** | **Amino acid** | **Coding** |
| --- | --- | --- |
| 1 | A | 10000000000000000000 |
| 2 | L | 01000000000000000000 |
| 3 | I | 00100000000000000000 |
| 4 | V | 00010000000000000000 |
| 5 | G | 00001000000000000000 |
| 6 | K | 00000100000000000000 |
| 7 | R | 00000010000000000000 |
| 8 | D | 00000001000000000000 |
| 9 | E | 00000000100000000000 |
| 10 | H | 00000000010000000000 |
| 11 | N | 00000000001000000000 |
| 12 | Q | 00000000000100000000 |
| 13 | S | 00000000000010000000 |
| 14 | T | 00000000000001000000 |
| 15 | C | 00000000000000100000 |
| 16 | M | 00000000000000010000 |
| 17 | Y | 00000000000000001000 |
| 18 | W | 00000000000000000100 |
| 19 | F | 00000000000000000010 |
| 20 | P | 00000000000000000001 |
